# Groups and Subgroups in Autism Spectrum Disorder (ASD) Considering an Advanced Integrative Model (AIM)

**DOI:** 10.3390/jpm14101031

**Published:** 2024-09-27

**Authors:** Andrés Ciolino, María Luján Ferreira, Nicolás Loyacono

**Affiliations:** 1 Planta Piloto de Ingeniería Química–PLAPIQUI (UNS–CONICET), Camino La Carrindanga Km 7, CC 717, Bahía Blanca 8000, Argentina; aciolino@plapiqui.edu.ar; 2 Departamento de Ingeniería Química, Universidad Nacional del Sur (UNS), Avda. Alem 1253, Cuerpo C’-Primer Piso, Bahía Blanca 8000, Argentina; 3 Departamento de Química, Universidad Nacional del Sur (UNS), Avda. Alem 1253, Bahía Blanca 8000, Argentina; 4 Sociedad Argentina de Neurodesarrollo y Trastornos Asociados (SANyTA), Migueletes 681, Piso 2, Departamento 2, Ciudad Autónoma de Buenos Aires C1426BUE, Argentina; nloyacono@sanyta.org

**Keywords:** ASD, concomitant medical problems (CMP), diagnosis, genetic model (GM), advanced integrative model (AIM), groups/subgroups

## Abstract

Background: Autism spectrum disorder (ASD) is related to social communication difficulties, repetitive behaviors, and highly restricted interests beginning early in life. Currently, ASD is more diagnosed than in the past, and new models are needed. The Advanced Integrative Model (AIM) is a new model in which genes and concomitant medical problems to diagnosis (CMPD) and the impact of their rigorous and adequate treatment are considered. Methods: The role of a dynamic encephalopathy from which the individual response, susceptibilities in the brain and outside the brain, gut barrier and brain–blood-barrier permeabilities, and the plastic nature of the brain is proposed as a tool for diagnosis. The concomitant medical problems (CMP) are those at and outside the brain. The individual response to treatments of CMP is analyzed. Results: The AIM allows for classification into 3 main groups and 24 subgroups. Conclusions: The groups and subgroups in ASD are obtained taking into account CMPD treatments and individual response.

## 1. Introduction

### 1.1. Autism Disorder Spectrum (ASD) Following the Advanced Integrative Model (AIM)

Autism spectrum disorder (ASD) applies to neurodevelopmental disorders characterized by early onset difficulties in communication and social skills and other symptoms [1]. When an ASD diagnosis is reported for an individual, the information that is given by physicians is that ASD is genetic and of unknown etiology. Generally, the concomitant medical problems to diagnosis (CMPD) are labeled as “comorbid” (that is, not related to ASD), and/or with no impact on the outcome.

The Advanced Integrative Model (AIM) is a new model of ASD with the Advanced Integrative Approach (AIA) in practice [2]. When a dynamic encephalopathy is considered in front of an ASD diagnosis, findings in subgroups are explained, even partially. Dr. Martha Herbert presented the model of ASD as a symptomatology of a chronic, dynamic, systemic encephalopathy and as a whole-body dysfunction [3,4], but it was not explored adequately. The encephalopathy would be dynamic considering the plastic characteristic of the brain [4]. The development of encephalopathy is then a process, not a genes-mediated fact for all people diagnosed with ASD, and may begin prenatally and/or postnatally.

In the following sections, we will present the application of an Advanced Integrative Approach (AIA) to ASD and the obtention of subgroups. The goal is to provide an overall approach to the diagnosis. This model is based on the division of ASD into three main groups, from which twelve subgroups per gender arise.

### 1.2. Explanation about Terminology

In the frame of an AIM, the ASD diagnosis is the name of emerging symptomatology [3]. In this sense, just after the ASD diagnosis, ASD is not considered a way of being, condition, or disease, but emerging symptoms. From here and thereafter in this manuscript, people diagnosed with ASD will be presented as such (children, teens, and adults): a child with ASD diagnosis and an adult with ASD diagnosis.

## 2. Methods

### 2.1. Literature Review

The literature search was carried out in Pubmed with selected keywords besides the word “autism”. They included prevalence, trajectories, heterogeneity, endophenotypes, treatment, regression, PANDAS/PANS, outcome, physical health, comorbidities, approach, model, subgroups, autoimmunity, biomarkers, and dysfunction. Other keywords were “care” and “quality of life”. Eighty-four articles were selected: 2 from 2024, 3 from 2023, 16 from 2022, 9 from 2021, 14 from 2020, 8 from 2019, 5 from 2018, 10 from 2017, 3 from 2016, and 14 from before 2016.

### 2.2. Main Groups in AIM/AIA

High levels of the so-called “comorbidity” have been reported by a systematic review [5], which concluded that “… medical comorbidity in children and adolescents with ASD and attention deficit hyperactivity disorder (ADHD) appears to occur in numerous medical areas…”. Bougeard et al. [6] reported that so-called ASD co-morbidities bring additional heterogeneity to the clinical presentation, which further advocates for personalized approaches to treatment and support.

In a recent manuscript from Hyman et al. [7], the recommendations for ASD after diagnosis were educational practices, developmental therapies, and behavioral interventions. CMPDs were not properly considered. Lyall et al. [8] pointed out the need for an integral approach to ASD in which genetic but also epigenetic and non-genetic factors should be considered. Yousef et al. [9] reported a community case study in Egypt in which the presence of factories near the house, children on medication during the first year of life, children with a chronic medical condition, children’s attachment to TV, particular medical conditions affecting the mother during pregnancy, and psychiatric disorders history in the family were considered as significant factors for the ASD prevalence. Biochemical factors such as folate metabolism abnormalities [10,11] or the increase in oxygen free radicals in metabolism due to abnormal bioprocessing of metal elements (Pb, Ba, Li, Hg, Cu, Se, and Zn) were also reported as important. Finally, Penzol et al. [12] reviewed the medical records of people admitted to the Comprehensive Medical Program for ASD (AMITEA) at the Gregorio Marañón University General Hospital from January 2012 to December 2015. Penzol. et al. discovered that almost one-third of them had at least one functional gastrointestinal disorder (fGID). Other reports presented a higher prevalence of GI disorders in ASD (more than 90%) [13].

Depending on the response to the treatment of the CMPD, different Main Groups in ASD may be envisioned [2]. The classification is based on the response of ASD symptoms to treatments of CMPD to ASD diagnosis and gives 3 big groups. The only group represented by the genetic (GM) or the neurodiversity (NM) models is the Main Group 3. In ASD, many neurological issues have links to outside-the-brain biological problems [14]. In particular, Dr. Frye et al. have published about CMPD from ASD [15,16,17,18], identifying them as such

Those works related to Pediatric Autoimmune Neuropsychiatric Disorders Associated with Streptococcal infections (PANDAS) [19], Pediatric Infection-triggered Neuropsychiatric Disorder (PITAND) [20], and Pediatric Autoimmune Neuropsychiatric Syndrome (PANS) [21] have demonstrated that these autoimmune issues may have behavioral and neuropsychiatric symptoms [22]. Therefore, PANDAS/PITAND/PANS symptoms are difficult to distinguish from ASD symptoms. In the AIM, we consider these medical issues as CMPD of ASD.

Recently, many manuscripts detect, count and report the so-called “comorbidities” [23,24,25,26]. There are two important references from 2024: one related to a Special Issue of *Frontiers in Psychiatry* [27], and the other is the study of a nationwide cohort [28]. CMPD to ASD is highly prevalent and widespread in people diagnosed with ASD. More and more manuscripts are being published about this topic.

According to the present paper, for Main Group 1, ASD symptoms would be only symptoms of a few CMPDs outside the brain affecting the brain (in a causal relationship to the encephalopathy?). For Main Group 2, ASD symptoms are the emerging symptoms of an encephalopathy related to multiple CMPD to ASD. Each CMPD “contributes” to the whole picture. Finally, for Main Group 3, ASD symptoms are not related to CMPD to ASD or do not respond to treatment of CMPD. In this case, they could be called “comorbid”.

If the CMPDs only contribute in a very complex and combined path to (and they do not “cause”) ASD, they cannot be studied separately. Today the link is known among the hypothalamus–pituitary–adrenal (HPA) axis, the gastrointestinal (GI) apparatus, the microbiota, and the immune system with the brain (the gut–microbiota–immune–HPA–brain axis). The individual response to the most rigorous, controlled, and serious allopathic treatments of CMPD of ASD gives clues to their role. Multimorbidity and complexity have to be considered. Therefore, the role of CMPD in ASD should be found AFTER and not BEFORE the adequate consideration of them. An additional component was missing in this picture: the analysis of the brain status in ASD.

## 3. Results

### 3.1. The Old and New Controversy: Static versus Dynamic Encephalopathy in ASD

The search for the genetic basis of ASD has been a topic of research since the manuscript from M. Rutter’s group [29]. Meanwhile, the prevalence has grown to 1 in 36 in 2020, according to the CDC data [30]. This value is related to the 1 in 44 previously reported [31] and to the near 1 in 20 males in children up to 17 years [32]. A recent manuscript reports a prevalence of 2.9% (2.4 to 3.6% in 95% CI) for children and adolescents in the USA aged 3–17 years old with ASD in 2019–2020 [33] or an average of 1 in 34 (with up to 1 in 28). The main point of the genetic model (GM) [34] is the consideration of the root of ASD as a static encephalopathy of prenatal origin. In the frame of the neurodiversity model (NM), ASD is a way of being [35].

We do know today a lot about ASD [36,37,38]. The findings in the results of treating CMPD to ASD cannot be fitted in the GM or the NM. CMPDs have been labeled as “comorbid” (do not change ASD). The reported improvements after their treatments have been assigned to coincidence. No genetic link in the brain to regression is found in ASD [39].

Up to 88% of children with ASD diagnosis have shown regression in prospective studies [40]. Regression is discussed and analyzed in the AIM as the end of a pre-encephalopathic, individual process and not a unique situation [41]. The dynamic encephalopathy related to ASD is assumed at the diagnostic step as chronic and systemic, a whole-body dysfunction associated with an encephalopathy in the AIM.

### 3.2. The Shift for the Role of CMPD of ASD from “Cause of” to “Contributor to”

A question that has been presented as important is what causes ASD for all people diagnosed with it. For the GM, the answer is genes, genes plus environment or genes and epigenetics plus environment at the prenatal step. The AIM gives a clearer answer. Without the complete characterization and knowledge of the complexity of ASD, any single hypothesis of causation is not well supported. There are thousands of manuscripts about different CMPDs of ASD outside the brain. Generally, they have been presented by looking only at one system or one parameter of a particular system at a time.

It is known that medical records for ASD are very incomplete. Many times, extensive biological exploration outside the brain in ASD does not exist. The information is available, but fragmented, disconnected. The question here is how many people diagnosed with ASD have CMPDs outside the brain? And how many people diagnosed with ASD have CMPDs outside the brain that are detected, diagnosed, and treated? These CMPDs are gut dysbiosis, food allergies, gastrointestinal issues and diseases (beyond celiac disease, i.e., non-celiac gluten sensitivity), nutritional, biochemical, and metabolic imbalances, mitochondrial dysfunction, endocrinological issues, oxidative stress, immune dysfunction, autoimmunity (folate receptor autoimmunity, PANDAS-PITAND-PANS, other), chronic infections (viruses, bacteria, parasites, and fungus), dysautonomia, toxic metals bioaccumulation, muscle diseases or disorders, connective tissues disorders or diseases, and motor and sensory integration issues, just to mention some. The clinical experience and the research show that many times a person with ASD diagnosis (child, teen or adult, male or female) has all or several of the above CMPDs in an individual combination. Therefore, the question for the AIM is as follows: “What are the medical problems contributing to an ASD diagnosis (emerging symptomatology) in this individual child (boy or girl), teen or adult (man or woman)?”

## 4. Discussion and Implications

### 4.1. Subgroups in ASD in an AIM

As we pointed out in Introduction, the AIM takes into account the role of CMPD in ASD. The response to CMPD treatment provides a tool to classify people with a diagnosis of ASD into twelve subgroups per sex. These subgroups can be obtained from the analysis of the three Main Groups in the AIM [2].

The proposed groups and subgroups are discussed as follows:

**Main Group 1**: With response of ASD core symptoms to the treatment of CMPD–Loss of diagnosis–ASD symptoms as symptoms of a totally Reversible Dynamic Encephalopathy related to CMPD. Examples: ASD secondary to celiac disease [42] without intellectual disability (ID). One subgroup.

**Main Group 2**

**Subgroup 2.1:** High response of ASD core symptoms to the treatment of CMPD and with/without ID–ASD as a symptom of a partially Reversible Dynamic Encephalopathy related to CMPD.

**Subgroup 2.2:** Moderate response of ASD core symptoms to the treatment of CMPD and with/without ID-ASD as a symptom of a partially Reversible Dynamic Encephalopathy related to CMPD.

**Subgroup 2.3:** Low response of ASD core symptoms to the treatment of CMPD and with/without ID-ASD as a symptom of a Dynamic partially Reversible Encephalopathy related to CMPD.

**Subgroup 2.4:** Very low response of ASD core symptoms to the treatment of CMPD and with/without ID-ASD as a symptom of a Dynamic partially Reversible Encephalopathy related to CMPD.

Therefore, we have here 16 subgroups (8 for males and 8 for females), by considering the ID properly diagnosed as the parameter to separate two additional categories by subgroup (with or without ID).

Examples: Recent literature on different treatments of CMPD and different responses to these treatments in different people of different ages with ASD in the cohorts [43,44,45,46,47].

**Main Group 3**

**Subgroup 3.1**: With no response of ASD core symptoms to the treatment of CMPD, without ID, no speech delay but ASD diagnosis with/without genetic correlation, and condition/way of being. Example: some adults with fewer support needs (ASD level 1 DSM-5 or Asperger syndrome (AS) following DSM-IV).

**Subgroup 3.2**: With no response of ASD core symptoms to the treatment of CMPD, with ID and speech delay, static encephalopathy with co-occurring genetic mutation, ASD symptoms present with a genetic disorder, and/or other disease/s present with the genetic disorder, called “Syndromic ASD” in the GM. Examples: some children with genetic disorders linked to ASD such as X Fragile, Angelman, Lesch Nyan, Landau Kleffner, Tuberous Sclerosis, Down’s syndrome, and others.

**Subgroup 3.3**: With no response of ASD core symptoms to the treatment of CMPD, with ID and speech delay, static encephalopathy without known co-occurring genetic mutation(s)/polymorphisms, and ASD symptoms of static encephalopathy of an unknown to date genetic disorder or other disease/s (infectious or not) or events (traumatic or not traumatic) with irreversible impact on the brain. Examples: ASD diagnosis after viral infection [48], bacterial infection [49], and traumatic brain event [50].

**Figure 1:**
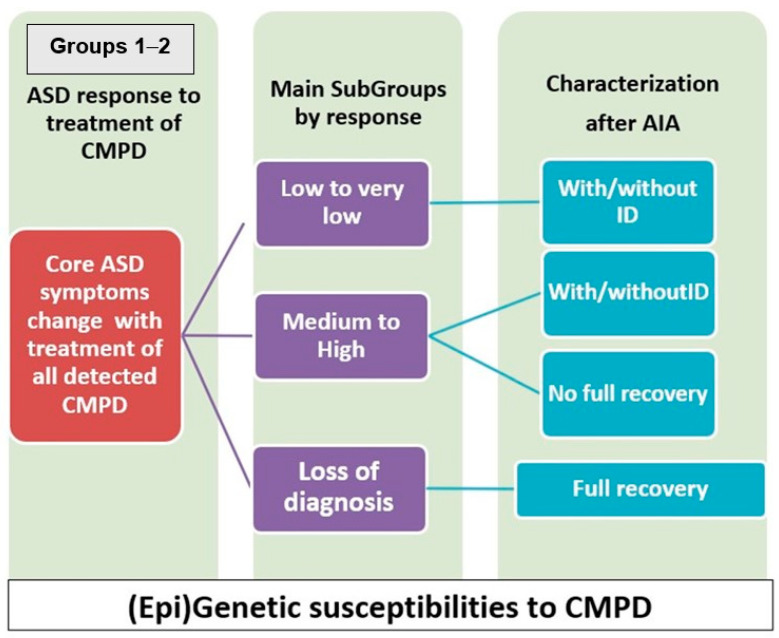
Main characteristics of Groups 1 and 2. AIA Advanced Integrative Approach.

**Figure 2:**
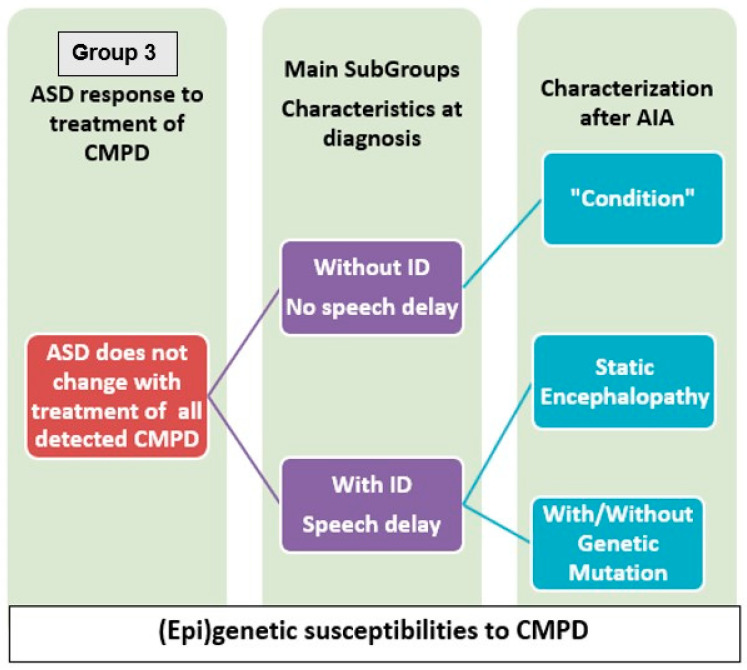
Main characteristics of Group 3. AIA Advanced Integrative Approach.

Figure 1 and Figure 2 summarize information about these twelve subgroups. A person diagnosed with ASD may begin in Main Group 2 and shift to Main Group 3 because of a lack of dynamism of the encephalopathy or change to irreversibility. In Main Group 2, it may also shift between subgroups, depending on the response to CMPD, or even shift to Main Group 1. Figure 1 presents the different subgroups taking into account the responses to treatment(s) of CMPD.

Former syndromic autism (even when present with chromosomic trisomy 21 such as in Down’s syndrome or with genetic ID such as X Fragile) could also be included in any of the three main groups related to ASD here presented by considering the response to the treatment of CMPD of ASD. Recent articles cite the improvement of ASD symptoms in animal models of X fragile with omega-3 fatty acids [51]. There is a case report of carbohydrate-specific diet with positive results in ASD/X Fragile syndrome—XFS [52].

Figure 2 shows how subgroups are defined when no response to treatment of CMPD is found. In the AIA, the focus is the detection of CMPD, the treatment, and the response to the treatment of CMPD of ASD, plus all the psychosocial therapies and supports needed at an individual level. No conclusion is achieved until all the detected CMPD are properly diagnosed and treated. A child with level 1 and another with level 2 of ASD (DSM-5) could respond similarly to the treatment of one CMPD. Response to treatments of CMPD, one per one, would be completely individual.

**Figure 3:**
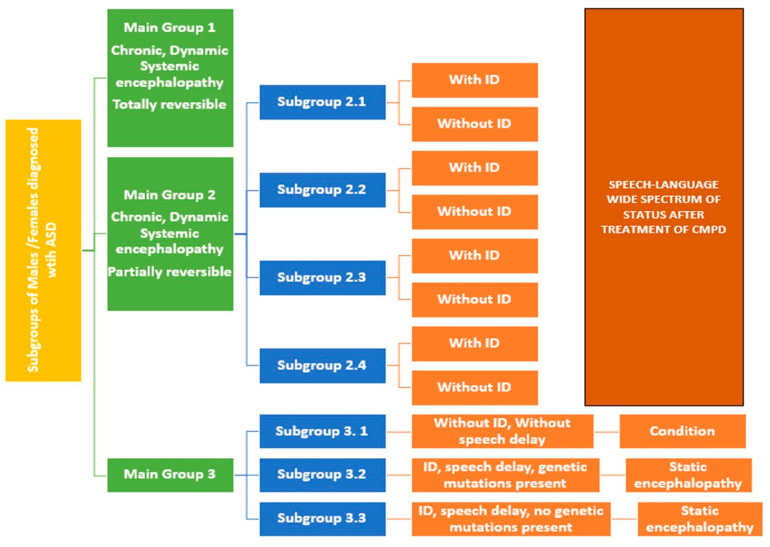
Complete presentation of groups and subgroups in an Advanced Integrative Model (AIM).

A longitudinal study of Asperger Syndrome (AS) (by the DSM-IV) reported that nearly 23 percent of adults with AS diagnosis had a restricted outcome with no occupation/activity and no friends, 47 percent had a fair outcome, and 27 percent had a good outcome [53]. Adults with ASD diagnosis without ID have higher rates of physical and psychiatric morbidity than the general population [54]. Treatment of gastrointestinal CMPD in AS not only changed symptoms of that CMPD, but also of AS itself [55]. AS has been reported to present chronic low detoxifying capacity for the last 10 years [56], as well as imbalances in omega-3/n-6 fatty acids [57]. Recent meetings have highlighted the health care in ASD [58], but the discussion about treatments for CMPD of ASD is biased by the genetic and neurodiversity models. Even more, the treatments of the CMPD of ASD are discussed as “treatments of ASD”. Figure 3 shows the 12 subgroups from the main 3 groups. The GM has been centered on the Main Group 3 and NM has focused on the subgroup 3.1 of the main group 3. Both models do not consider Groups 1 and 2, and proposed the research about the prenatal, genetic links to ASD (and now the social and services supports are lifelong) instead of also looking at the CMPD to ASD and the incredibly important (and enormous) amount of information that can guide the design of better-updated research in ASD.

### 4.2. And Speech/Language?

Different patterns of language are found in children diagnosed with ASD. Recent studies have demonstrated that some biologically based approaches to the whole-body dysfunction in ASD promote the development or the further evolution of speech/language [59]. There are different proposals about the impact of whole-body dysfunction in ASD [60]). More than a decade ago, it was published that about 187 children with verbal apraxia responded to vitamin E plus polyunsaturated fatty acid supplementation [61]. A recent article reported that treatment for children with ASD and low verbal ability has been neglected historically [62]. Finally, it is important to emphasize that at each subgroup, a wide spectrum of speech outcomes can be found, even after improvements in whole-body health.

### 4.3. The Dynamic Character of ASD: Changes in Trajectories in ASD

ASD is a dynamic diagnosis in the first years of life. Fountain et al. [63] presented six different trajectories in 6975 children aged 2 to 14 years using 9 evaluative items. Six typical patterns of social, communication, and repetitive behavior functioning, which were very heterogeneous, were identified. The studies from Georgiades et al. [64] examined the trajectories of autistic symptoms in 187 children with ASD evaluated across four time points from diagnosis to age 10. Trajectories were labeled as Continuously Improving (27%), and Improving then Plateauing (73% of the sample). There were differences in symptom severity, language, cognitive, and adaptive functioning skills. In another study from Waizbard-Bartov et al. [65] with 125 children diagnosed with autism spectrum disorder (ASD) and assessed at approximately 3 and 6 years of age for autism symptom severity, IQ and adaptive functioning found a Decreased Severity Group (28.8%); a Stable Severity Group (54.4%) changed by 1 point or less; and an Increased Severity Group (16.8%) increased by 2 or more points. There was no clear relationship between intervention history and membership in the groups.

In the study of Simonoff et al. [66], 158 participants with ASD were evaluated at 12 years and 126 (80%) were reassessed at 23 years. Several tests were used (Wechsler Intelligence Scale for Children (WISC), Wechsler Abbreviated Scale of Intelligence (WASI and WASI-2), VABS (Vineland Adaptative Behavior Scales), ABAS (Adaptive Behavior Assessment System), Social Responsiveness Scale (SRS), and more. The authors found an unexpected mean significant IQ increase of 7.48 points but the number of ASD symptoms remained stable over time. No information about CMPD was provided.

Two hundred and three children were referred for ASD evaluation at ages 1 to 4 years and assessed at three time points at intervals ranging from 9 months to 3 years. Assessments included Autism Diagnostic Observation Scale (ADOS), language (ADOS-language item), nonverbal IQ (NV-IQ; different tests adequate to chronological/mental age), and parent-reported behavioral problems (Infant–Toddler Social and Emotional Assessment, Child Behavior Checklist). Analysis with ADOS total scores led to the identification of three main stable and two small improving groups. A severe–stable group (19.5% of the sample) showed persistent low NV-IQ and a marked increase in attention problems over time. A moderate–stable group (21.7%) (with below-average increasing Non-Verbal (NV)-IQ) was detected. A mild–stable group (48%) with stable–average NV-IQ and the highest scores on attention deficit hyperactivity disorder (ADHD)-related traits had increased ASD symptoms despite stable–low ASD scores. Two groups (each 5.4%) improved. One moved from severe to moderate ASD scores, and the other moved from moderate to mild/no spectrum scores. Both of these groups improved in language, NV-IQ, and ADHD-related traits [67]. The phenomenological–social descriptive approach is prevalent [68], but no CMPDs are taken into account.

Of the 76 adults with ASD from France [69], two-thirds of them with severe autism had a very poor outcome. The trajectories of people with AS (DSM-IV), or for those with less support needs, were better but remained dependent on aging parents who had few available supports. Systematic reviews about outcomes show that the long-term outcome of almost half of all adults with ASD is poor, 31.1% is fair and only 19.7% is good [70].

Cawthorpe [71] published about the number of International Classification of Diagnosis (ICD) in ASD. Males with ASD had an overall higher than 1 odd ratio (OR) in 11 main ICD-9 classes. Females with ASD had higher ORs (>1.0) in 12 main ICD-9 classes. The importance of physical health has been highlighted [72]. A manuscript from 2018 [73] found that for males with ASD over a 16-year period, the odd ratios (OR) were significantly greater than 1 for 15 of the 17 main ICD classes and 10 of the main ICD classes for females. Twenty-eight (28) ICD disorders significantly preceded and 95 ICD disorders significantly followed ASD for females in a population of people diagnosed with ASD. Thirty-eight ICD disorders significantly preceded and 234 ICD disorders significantly followed ASD for males. People with ASD diagnosis have multiple chronic health conditions and die prematurely [74], especially if they have an intellectual disability. Sixty three ASD plus intellectual disability adults were assigned to groups considering their multimorbidity [74]. They identified four classes of participants. They were distinguished by their multimorbidity status, independence, and number of treatments. The authors say “… These findings support the hypothesis that an altered gut–brain relationship is involved in the risk of autism spectrum disorder, its outcome, and its association with chronic health conditions…”. The true multimorbidities in physical health outside the brain remain without evaluation, diagnosis, and treatment. The potential for adverse effects of psychiatric medication is unknown, especially in front of polypharmacy.

The recent special issue of the Irish Journal of Psychological Medicine and the manuscript of Gallagher & Grath [75] presented that mental health services are not currently meeting the needs of autistic people across the lifespan. The authors emphasized that under-recognized co-occurring conditions can contribute to less optimal outcomes. No efforts have been made to include the CMP (besides neurological and psychiatric ones) in the long-term outcome analysis from childhood to adulthood. The efforts to subgroup ASD using different criteria have been unsuccessful up to date [76]. The need for new approaches is reported [77] and new subgrouping criteria have been presented [17].

CMPD to ASD in infancy and adulthood have a higher prevalence than the general population and multi- or hypermorbidity has been reported. However, these CMPDs have been systematically overlooked, underdiagnosed, and worse, untreated, for whole cohorts of children, teens, and adults (men and women) diagnosed with ASD. Sometimes this situation is the norm (for years or decades). There are several voices asking for a profound change in how we look at and approach ASD as a diagnosis [78]. A dynamic and systemic approach is urgently needed [5,72,79,80,81]. A new approach, the AIM and the AIA, to ASD have been presented and explained elsewhere [2]. The possibility of an outcome when the diagnosis was correctly given and later the person does not fit the criteria has been published. Eigsti, Fein & Larson proposed “loss of autism diagnosis” for this situation rather than “optimal outcome” in ASD [81].

Three main findings support the AIM for ASD, as follows:

a The number, severity, and complex combination of CMPD reported in mainly descriptive studies in children, teens, and adults with ASD. These CMPDs include those in the brain (correlated with neurological and psychiatric diagnosis from the DSM) and outside the brain (correlated to whole-body dysfunction).
b The high-quality scientific evidence, academic reports from universities/ONGs, and professionals in consultation practice around the world of changes in multiple symptoms of ASD upon the treatment of CMPD of ASD in children, teens, and adults. There are multiple case reports available and some studies with multiple treatments of CMPD at once.
c The increasing number of scientific reports about the nature of the CMPD of ASD in different groups of people with ASD diagnosis.

## 5. Conclusions

The response to the treatments of CMPD of ASD allows defining subgroups in ASD. The CMPD may be comorbid, contributing, or causal to the underlying biological link to the emerging symptomatology in ASD. Without the information about CMPD outside the brain present in each person diagnosed with ASD from before diagnosis and later in prospective studies, the research design and analysis will always be incomplete. New ideas such as the system biology and whole-body dysfunction applied to ASD have to be discussed. It is difficult due to the framework of the GM and the close dogmatism that sometimes is present in academic and professional fields. There is space for neurodiversity, genetics, and even the social model of disability in a truly complete, bio-psychosocial model of ASD: the AIM. The subgroups in ASD are found after the treatments of CMPD, considering the individual answers to them. With the application of the AIM, up to 24 subgroups can be obtained (12 for males and 12 for females).

Forthcoming manuscripts will deal with the objective metrics of outcomes and the response to treatments of CMPD of ASD for the different subgroups.

## Data Availability

No new data were created or analyzed in this study. The article presents a new point of view about how to obtain subgroups in ASD, considering published literature from other authors and of our own. It is based on a literature review but the manuscript is not a review paper. This is the reason of the selection of the classification "Perspective". When considering the final Subgrouping depending on response, the manuscript presents a new procedure to obtain Subgroups in ASD. Due to the nature of the manuscript, it depends on the point of view you may answer differently the first question.
